# Life cycle contributions of copper from vessel painting and maintenance activities

**DOI:** 10.1080/08927014.2013.841891

**Published:** 2013-07-11

**Authors:** Patrick J. Earley, Brandon L. Swope, Katherine Barbeau, Randelle Bundy, Janessa A. McDonald, Ignacio Rivera-Duarte

**Affiliations:** a SPAWAR Systems Center Pacific, San Diego, CA, USA; b Scripps Institution of Oceanography/UC San Diego, La Jolla, CA, USA

**Keywords:** copper antifouling paint, cleaning, loading, toxicity, leach rate, life cycle contributions

## Abstract

Copper-based epoxy and ablative antifouling painted panels were exposed in natural seawater to evaluate environmental loading parameters. In situ loading factors including initial exposure, passive leaching, and surface refreshment were measured utilizing two protocols developed by the US Navy: the dome method and the in-water hull cleaning sampling method. Cleaning techniques investigated included a soft-pile carpet and a medium duty 3M™ pad for fouling removal. Results show that the passive leach rates of copper peaked three days after both initial deployment and cleaning events (CEs), followed by a rapid decrease over about 15 days and a slow approach to asymptotic levels on approximately day 30. Additionally, copper was more bioavailable during a CE in comparison to the passive leaching that immediately followed. A paint life cycle model quantifying annual copper loading estimates for each paint and cleaning method based on a three-year cycle of painting, episodic cleaning, and passive leaching is presented.

## Introduction

Preventing fouling on vessels can be traced back to the fifth-century B.C. and earlier, with efforts primarily focused on hull preservation, water resistance, and maintaining vessel speed (WHOI 1952). As modern day technology has developed, the benefits associated with a well-conditioned hull and the prevention of biofouling have increased to include considerations associated with the entire life cycle, operating and support costs critical to vessel operations (Copisarow 1945; Schultz et al. 2011). The most common fouling solution in use involves the application of a paint system consisting of a primer/base coat and a compatible copper-based antifouling (AF) coating on the underwater portion of a vessel. Copper released from the coating acts as a toxicant inhibiting the settling and growth of marine organisms on the coated surface. For recreational vessels in harbors and marinas throughout California, the two most commonly applied paint types are ablative and epoxy-based coatings that utilize different physical and mechanical properties to release copper to reduce biofouling. During the life cycle of a coating, biofouling often appears and requires cleaning to maintain a smooth, fouling-free surface (Schiff et al. 2004; Port of San Diego 2006, 2011). Underwater cleaning practices in California follow industry standards utilizing progressively aggressive methods and tools depending upon the condition of the hull, ranging from microfiber cloth and soft pile carpet to varying roughness of 3M™ type abrasive pads to remove fouling (CPDA 2008). Cleaning often occurs every four weeks during low fouling conditions (eg winter months) and every three weeks during high fouling conditions (eg summer months) (Port of San Diego 2011). Because of the widespread use of copper-based AF coatings and other anthropogenic loadings, copper concentrations in some harbors exceed or are near the United States Environmental Protection Agency (USEPA) water quality criteria (Port of San Diego 2011).

Copper levels exceeding water quality criteria have triggered regulatory controls such as Total Maximum Daily Loads and National Pollution Discharge Elimination System permit restrictions limiting the loading and concentrations of copper in harbors and particularly yacht basins and marinas (Port of San Diego 2011). Environmental loading from AF paint systems includes contributions from passive hull leachate and surface refreshment (SR) of the paint. Passive hull leachate is the continuous dissolution of AF paint constituents during environmental exposure. SR includes cleaning activities and vessel operation that refresh the paint surface through active removal of fouling organisms and varying amounts of AF paint. These loading components are interdependent because SR affects passive leach rate (at least temporarily), and the magnitude of the passive leach rate is a key factor in influencing the level of antifoulant released to the surrounding aquatic environment. Conversely, as fouling increases due to reduced coating efficacy, the demand for SR increases.

SR activities and passive leaching may contribute to ambient water toxicity. Once released from a coating, copper hydrates and/or complexes with a variety of species in natural seawater environments, including the hydrated free copper ion (Cu^2+^), dissolved organic (labile and inert) and inorganic complexes, colloidal and particulate copper (Morel 1983). Ligands, humic substances, and related compounds associated with the surface of the biofilm may be present in sufficient quantity in the surrounding water to complex the copper and make it less bioavailable. Copper has been shown to be strongly associated with organic matter (ligands) in the marine environment (Campos & van den Berg 1994), and these copper–ligand complexes are less labile and therefore less toxic than the uncomplexed form (Cu^2+^, referred herein as free copper). Thus, high concentrations of organic copper-binding ligands in coastal estuaries have been shown to effectively buffer copper toxicity even at relatively high copper loadings (Buck & Bruland 2005; Rivera-Duarte et al. 2005). Copper concentrations that exceed the binding capacity of the natural ligands can lead to potentially toxic copper conditions, generally thought to occur at [Cu^2+^] > 10^−11^ M (or log[Cu^2+^] > ^−11^; Brand et al. 1986; Rivera-Duarte et al. 2005). While this toxicity is a function of the effectiveness of the individual coating, the aggregate release from many vessels to the environment must also be considered; especially for harbors and marinas where large concentrations of vessels occur and water circulation may be limited (Schiff et al. 2004).

Standard methods for release rate determination (ASTM 2006, 2007; ISO 2007a, 2007b) are useful for gaging the effectiveness of paint systems. However, it is widely recognized that these methods do not produce data representative of in-service conditions, do not reflect environmental release rates for AF products, and are not suitable for deriving environmental loading estimates (Schiff et al. 2004; ASTM 2006, 2007; Finnie 2006). The US Navy's Dome method was utilized during this study as it is considered the most reliable method to date for determining actual biocide release rates from AF paints (Finnie 2006; OECD 2012).

Previous studies have examined environmental copper loading in situ associated with passive leaching from vessels (Seligman et al. 2001; Schiff et al. 2004) static and dynamic cycles (Lindner 1993), as well as loading associated with cleaning activities (Schiff et al. 2004; Port of San Diego 2006). The objective of this study was to develop a life cycle model for environmental loading associated with AF coatings including passive leachate and SR activities. The experimental design was based upon integrating elements from these previous studies to validate the model and provide a more comprehensive dataset, and better resolution of release rate and loading dynamics.

## Materials and methods

### Field methods

Two copper-based AF paints were evaluated in this study, both containing cuprous oxide as the active ingredient to prevent fouling. The paints tested were representative of the most commonly utilized paints for recreational boats in California (Port of San Diego 2006). The first paint tested was an ablative paint containing 38% cupric oxide (wet weight), formulated with a controlled solubility copolymer designed to wear away over time to provide a fresh biocide at the surface of the coating. The second paint evaluated was an epoxy-based coating containing 65% cuprous (wet weight), formulated with a hard insoluble matrix to slowly release biocide via a diffusion-controlled process.

Paints were applied to 45 cm × 45 cm × 0.3 cm fiberglass panels. The panels were laid out, wiped free of contaminants, primed, and painted. Four coats of paint were applied to the front and back of each panel, achieving a uniform 2 mm dry film thick coverage, based on the wet-weight of the paint. Once dry, the panels were secured to fiberglass racks and exposed to natural seawater conditions for 106 days at the SPAWAR Systems Center (SSC)-Pacific Harbor Research Platform System deployed in San Diego Bay, California (32°42′19.28″N, 117°14′10.08″W). Each rack contained two test panels deployed vertically below a 1 m depth facing NNW. Each rack was configured with a single replicate of each coating and the position of the replicates within the water column varied across the racks. Passive leaching of total and dissolved copper was measured utilizing the SSC Dome technique (Seligman & Neumeister 1983) and the in-water hull cleaning sampling method was used to measure the particulate loading associated with cleaning activities (Chadwick et al. 2008). A high number of dome deployments (n = 192), samples (n = 1580), replicates (n = 3), and strict laboratory processes and clean sample handling procedures were utilized throughout the study to understand variability and increase confidence in the results. Test paints were evaluated under three different cleaning scenarios, with each scenario performed in triplicate, totaling 18 panels. The first scenario was no-cleaning (untreated); the second scenario was cleaning with a soft-pile carpet, representing the Best Management Practice (BMP) (CPDA 2008); and the third scenario was cleaning with a Scotch-Brite™ (ID #70071592383) medium duty general purpose scouring pad (3M™ pad) that represents a more aggressive, non-BMP, cleaning method (CPDA 2008). The latter two cleaning scenarios were applied after exposure for 60 days because higher release rates are associated with newly applied coatings and cleaning is not required during this initial exposure (IE) period (CPDA 2008). Total and dissolved passive leach rates of copper were measured at various time points throughout the course of the experiment with the in situ dome system (Seligman & Neumeister 1983; Valkirs et al. 2003). To maximize fouling, all panels were deployed during peak summer fouling conditions on 13 August 2012. Untreated panels were sampled on 12 separate events, starting from 1 h after the materials were deployed, followed by events on days 1, 2, 3, 5, 7, 15, 30, 45, 59, 60, and 90. Treatment panels were deployed concurrently with the untreated panels, with the evaluation of the first passive leach rate taking place on day 59. Panels were then cleaned using one of the two treatment methods (BMP or non-BMP) on day 60. To quantify the dissolved and particulate masses released during the cleaning events (CEs), three representative areas from each panel were cleaned using the in-water hull cleaning sampling device outfitted with the designated treatment method (ie BMP or non-BMP) (Chadwick et al. 2008). The remaining area of the panel surface was manually cleaned using the corresponding method. Measurements of the passive leach rate with the dome system were taken from the treatment panels at nine additional intervals, starting 1 h after the cleaning and followed by measurements on days 61, 62, 63, 65, 67, 75, 90, and 106.

The dome system and method developed by the US Navy (Seligman & Neumeister 1983) and described in detail by Seligman et al. (2001) and Valkirs et al. (2003) were used for evaluating passive leach rates. This system isolates a volume of ambient water over a surface and provides water circulation in a closed loop. The system allows for a confined volume of water to be exposed to the effects of leaching from a surface area with small aliquots withdrawn at regular intervals. Five aliquots of 50 ml were withdrawn from the dome at 15 min intervals (0, 15, 30, 45, and 60 min). Approximately 25 ml of each sample were filtered through a 0.45 μm disc filter, while the remaining 25 ml were unfiltered, representing the dissolved and total fractions respectively. Samples were acidified to pH ≤2 with quartz still-grade nitric acid (Q-HNO_3_) in a High Efficiency Particle Air class-100 all polypropylene working area.

Additional samples were taken for determination of the free copper ion (Cu^2+^) at selected time points during the course of the study (day 1, 60, and 61). To evaluate system sensitivity, on day 1, samples were collected both inside the dome device (referred to as ‘dome’ samples herein) and immediately adjacent to the dome (referred to as ‘proximity’ samples herein). Samples were taken using a peristaltic pump with acid-washed Teflon and C-flex tubing that was rinsed with ∼ 2 1 of ambient sea-water before sampling. Samples were filtered in-line with a 0.2 μm Acropak™ 200 capsule filter into acid-cleaned 500 ml FLPE bottles, placed in a cooler with ice packs and frozen at −20 °C at the laboratory (within 4 h). In order to determine the ambient copper organic speciation at the time of the study, on days 1 and 60, samples were collected every 2 h ∼ 20 m away from the study site. Dissolved organic carbon (DOC) samples were collected concurrently with ambient samples using the same technique as for the dissolved copper organic speciation samples. DOC samples were immediately acidified with two drops of concentrated trace metal grade hydrochloric acid (HCl).

**Figure 1.**
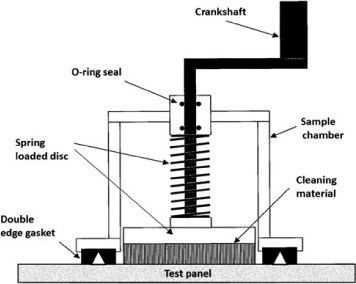
Hull cleaning sampling chamber.

During the CE on day 60, each test panel was tested in triplicate utilizing the in-water hull cleaning sampling method (Chadwick et al. 2008). This method stipulates the use of a device that simulates a hull cleaning and provides standardization for in situ sampling of naturally biofouled surfaces capturing dissolved and particulate matter. The hull cleaning device consists of a clear polycarbonate cylinder, with an inside diameter of 11.4 cm, a sampling area of 101.6 cm^2^, and a sample volume of 1575 ml. The cylinder opening has an integrated double edge gasket to seal against the test surface. On the opposite end of the cylinder, a shaft passes through an O-ring seal in a polycarbonate cap and attaches to a spring-loaded disk inside the cylinder ensuring constant pressure during sampling activities (Figure 1). The disc accommodates the use of any cleaning material (carpet, 3M™ pad), and an exterior handle on the shaft allows the disc to be manually rotated for a set number of revolutions. To simulate cleaning, one revolution was determined to provide sufficient biofouling removal without damaging the coating.

Prior to each CE, new cleaning discs were stored in high-purity (18 MΩ cm^−1^) water. Discs were installed in the hull cleaning device, the spring was retracted, and to reduce interference, the cylinder was pre-filled with 0.45 micron-filtered San Diego Bay seawater and temporarily sealed with a clean polycarbonate sheet. The hull cleaning device was held ∼ 1 cm above an undisturbed fouled area on the test panel. The polycarbonate sheet was removed and the hull cleaning device was sealed against the test panel and held in place to ensure seal integrity. The interior spring was released applying consistent pressure between the cleaning disc and the test panel surface. The shaft was rotated one turn at ∼ 10–15 rpm. The spring was retracted and the polycarbonate sheet was slid between the test panel and the cylinder gasket (ensuring that the sheet did not contact the panel surface) capturing the seawater and associated particulate matter. The hull cleaning device was taken out of the water and a 25 ml aliquot was removed and filtered through a 0.45 μm disc filter, representing the dissolved component of the CE. The cleaning pad and sampling apparatus were rinsed with 0.45 μm-filtered seawater to wash off all particulates. The sample and associated equipment rinse water were decanted into a nonacidified plastic 2-l sample bottle and all samples were stored at 4 °C until further processing. Between CEs, the hull cleaning device was rinsed twice with ambient seawater and once with 0.45 micron-filtered seawater.

### Laboratory methods

#### Metal concentration quantification

Quantification of metal concentrations followed trace metal clean sampling techniques throughout the collection, handling, and analysis (USEPA 1996). Metal concentrations were measured with a Perkin-Elmer™ SCIEX ELAN DRC II inductively coupled plasma with detection by mass spectrometry (ICP-MS; USEPA 1994). As necessary, to minimize matrix-related interference, samples were diluted with 0.1 N Q-HNO_3_ made up in 18 MΩ cm^−1^ water. The diluted samples were injected directly into the ICP-MS via a Perkin-Elmer™ Autosam-pler 100, while the undiluted samples were preconcentrat-ed with a Perkin-Elmer™ FIAS 400 flow injection system, following the methodology presented by Ndung'u et al. (2003), and a Toyopearl™ AF-Chelate-650M resin from Tosoh Co. Analytical standards were made with Perkin-Elmer™ multi-element standard solution (PEMES-3) diluted in matrix-matched 1 N Q-HNO_3_, and were analyzed at the beginning and end of each run. The analysis also included measurement of the Standard Reference Material (SRM) 1643e with recoveries within 15%. Replicate samples had a coefficient of variation of ≤5%. The method limit of detection, defined as three times the standard deviation (SD) of the procedural blanks made of 1 N Q-HNO_3_, averaged 0.21 + 0.20 μg 1^−1^.

Dissolved copper speciation samples were gently thawed at −4 °C (over two days) and shaken vigorously before analysis. Sample aliquots of 10 ml were placed into 10 separate acid-washed Teflon cups (Savillex™). Two of the Teflon cups contained no added copper, and the remaining eight cups contained 1, 2.5, 5, 8, 15, 25, 50, and 100 nM copper (0.06, 0.16, 0.32, 0.51, 0.95, 1.59, 3.18, and 6.36 μg 1^−1^) to titrate the naturally occurring organic copper-binding ligands. Each cup was run using competitive ligand exchange adsorptive cathodic stripping voltammetry (Buck & Bruland 2005) with salicylaldoxime (SA) as the added ligand. The titration data were analyzed using the average and SD of a combination of van den Berg-Ružić linearizations (Ruzic 1982; van den Berg 1982) and Scatchard linearizations (Scatchard 1949; Mantoura & Riley 1975) to determine the copper-binding ligand concentrations (L) and their conditional stability constants (log *K*_CuL, Cu^2+^_^cond^). The concentration of Cu^2+^ (expressed as log [Cu^2^]) was calculated from the total dissolved copper concentrations determined initially within the dome system (*t* = 0) and the copper-binding ligand data according to Moffett and Dupont (2007). Additional Cu^2+^ concentrations were calculated with the ligand data and the dissolved copper concentrations determined from ambient (pier) samples. Three separate titrations were completed for each sample, with two different concentrations of the added ligand SA (5 and 25 μM). The data presented here are from the titrations completed with 5 μM SA, as this was determined to be the appropriate competition strength given the concentration and strength of the ligands detected (determined logα_Fe(SA)_2__/logα_Fe(L)_*x*__; Donat & van den Berg 1992). Titrations done at the highest concentration of SA (highest analytical window) were used as an overload ‘titration’ to verify the internally calibrated sensitivity (Kogut & Voelker 2001).

For the CE, filtered samples and the particles retained by the filters were analyzed for metal loading. The 1.5 1 samples collected using the in-water hull cleaning sampling method during the CE at day 60 were filtered in the laboratory on dried, pre-weighed 0.45 μm Nucle-pore™ filters. An aliquot of the filtered sample was collected and acidified for ICP-MS analysis. Multiple filters were used for filtration of the whole volume, then dried at 30 °C overnight and reweighed. Filters were subsequently digested for analysis of total copper concentration based on method 140.0 for the analysis of trace metals in marine sediments (NOAA 1998). The limit of detection and reporting for the digestion of the particles is 0.62 μg g^−1^ based on six replicate digestion blanks. The acid digestion of the particles was designed to represent the metal associated with the surface of the particles. Therefore, it did not entirely disintegrate the mineral particles, resulting in lower recoveries of SRMs. This was evidenced in the recoveries of the sediment SRMs included in the digestion. Three separate SRMs were analyzed in triplicate with the set of samples; these are the marine sediments BCSS-1, MESS-2, and PACS-1. Recoveries measured for these SRMs were 67, 74, and 65%, respectively, which is a narrow range, in spite of the fact that they cover an order of magnitude in copper concentration range (18.5, 39.3, and 452 μg g^−1^, respectively).

#### Leaching rate calculations

Leach rate estimates were derived from the time variants of individual ICP-MS copper concentrations for each dome deployment. Initial dome volumes were approximately 2.8 1. Volumes were corrected for water removed throughout the 1 h testing period. The measured copper concentration multiplied by the system volume equals the total Cu mass in the system. These mass values were then regressed against time (samples were taken at 0, 15, 30, 45, and 60 min) to calculate a daily release rate using the slope of the fitted line. Values were normalized to the surface area of the test panel covered by the dome resulting in a final Cu release rate in μg cm^−2^ d^−1^. For each set of dome deployments, copper release over time was linear with *R*^2^ values from all regressions ≥0.95.

#### Cumulative copper loading

The cumulative copper loading (CL) over a given time interval (*x*_0_, *x*_*n*_) can be approximated from the measurements of leach rate (R) using the following equation:


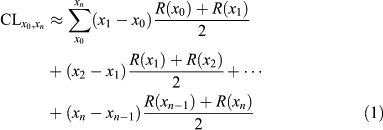


where,

CL_*x*_0_;*x*_*n*__ = the cumulative copper loading (μg cm^−2^) from day *x*_0_ through *x*_*n*_.

*x*_0_, *x*_*n*_ = a series of consecutive time points (days) during which measurements of release rate were made beginning with day *x*_0_ and ending with day *x*_*n*_. For example, untreated panels were sampled on 12 separate events, starting from 1 h after the materials were deployed (0.042 day or 1/24 day), followed by events on days 1, 2, 3, 5, 7, 15, 30, 45, 59, 60, and 90.

*R*(*x*_*n*_) = the measured release rate (μg cm^−2^ d^−1^) for time point *x*_*n*_.

With respect to the curves of AF release rate, care should be taken to have an adequate number of sampling points early in the generation of curve (two to three sampling events each week for the first two weeks) to accurately capture the shape of the curve during this highly dynamic period.

#### Paint life cycle loading estimates

Copper loading estimates over the course of the paint life cycle were made by combining leach rate data and cleaning data. Four different loading variables were established based on a combination of leach rate dynamics, coating performance, and boat usage factors: (1) IE: Cumulative copper released as leachate due to IE of a newly painted boat. Typically, throughout the industry, boats are not cleaned for a certain amount of time following new painting because the higher leach rates preclude the requirement for cleaning (CPDA 2008). For this study, IE is equal to the cumulative copper release from the untreated panels for the first 60 days, where IE = CL_0,60_ untreated panels. (2) Pseudo-Steady State (PSS): A PSS is an average release rate (μg cm^−2^ d^−1^) from hull leachate over a period of time when rates have stabilized to an asymptotic low. Specifically, this is at least 24 days and contains four or more data points where the arithmetic mean between two consecutive points differs from the final calculated weighted mean release by not > 15% (ASTM 2006). Building from Equation 1, the PSS can be calculated by the following:


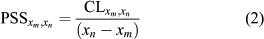


(3) SR: Any action that refreshes the paint surface resulting in particulate release and/or a subsequent increase in leachate. This may include hull cleaning activities (SR_CE_) or normal boat usage where fouling is removed while the boat is underway (SR_B_). The SR values represent dissolved and particulate copper released as a direct result of the activity. For this study, SR_CE_ represents dissolved and particulate copper released as a direct result from BMP and non-BMP cleaning activities collected using the hull cleaning sampling chamber (Figure 1). (4) Leachate (L): Cumulative copper release due to leachate following a SR event. The L value is unique to the SR type, and the duration used to calculate L depends on the frequency of the SR event. For example, L_CE_*n*__ is the cumulative release for *n* days following a CE, after which another SR event would occur or a PSS is established. Similarly, L_B_*n*__ is the cumulative release for *n* days following boat usage, after which another SR event would occur or a PSS is established.

The total copper loading based on the life cycle of a paint can be estimated using the above variables with the following equation:


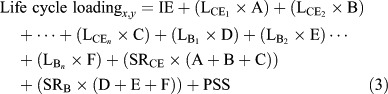


where,

Life cycle loading_*x, y*_ = cummulative copper release (μg cm^−2^) between time points *x* and *y*. Days *x, y* represent the time interval between hull painting during which the coating is exposed to water.

L_CE1_; L_CE2_; L_CE_n__ = L following regularly scheduled CEs between different time intervals CE_1_, CE_2_, … CE_*n*_.

L_B1_; L_B2_; L_B_*n*__ = L following boat usage between time intervals B_1_, B_2_, … B_*n*_.

A, B, C = the number of times events L_CE1_; L_CE2_; L_CE_*n*__, occur between days *x* and *y*.

D, E, F = the number of time events L_B1_; L_B2_; L_B_*n*__ occur between days *x* and *y*.

For this study, several assumptions were made to calculate the life cycle loading: the time between vessel painting was based on a typical paint life cycle of three years. Only SR related to CEs was considered SR_CE_ and did not consider boat usage because that was outside the scope of the original project. Two cleaning frequencies were established based on the average cleaning schedule in San Diego Bay (Port of San Diego 2011) of every three weeks (21 days) during the summer months of June, July, and August and every four weeks (28 days) during non-summer months, resulting in L_CE21_ and L_CE28_. This study established that the coating was applied during the winter, resulting in 12 CEs during the first year (8 nonsummer, and 4 summer) and 14 CEs during the subsequent years (10 non-summer and 4 summer). The magnitude of copper release related to the individual CEs did not decrease with time. A PSS was never reached because of the frequency of CEs. Therefore, the estimated life cycle loading is:


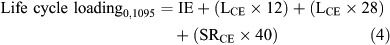


**Figure 2.**
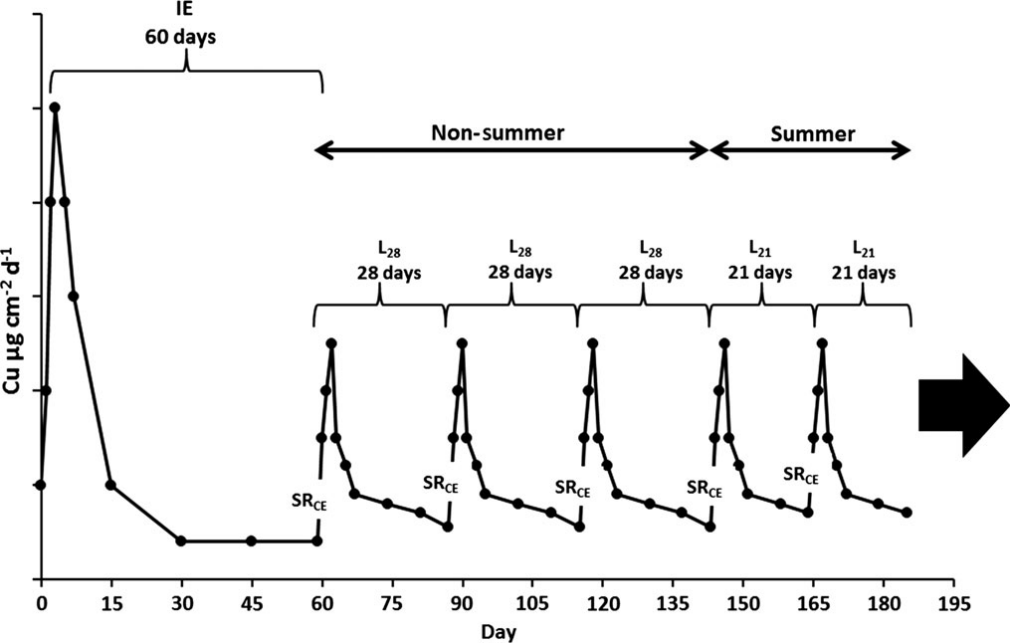
Graphical representation of leach rates under different loading scenarios: IE, 28 and 21-day life cycle loading (L_CE28_, L_CE21_), and SR from cleaning activities (SR_CE_).

A graphical representation of the loading scenario is presented in Figure 2.

Additionally, a loading scenario was calculated based on no SR events, assuming no CEs and no boat movement. This scenario was considered unrealistic, but represented a minimum loading threshold value for the coating. The loading scenario was based on release rates during the IE period and the PSS release rate. The no cleaning/no movement loading was calculated as:


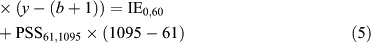


## Results

### In situ leach rates of copper and loading inputs

#### Initial exposure

**Figure 3.**
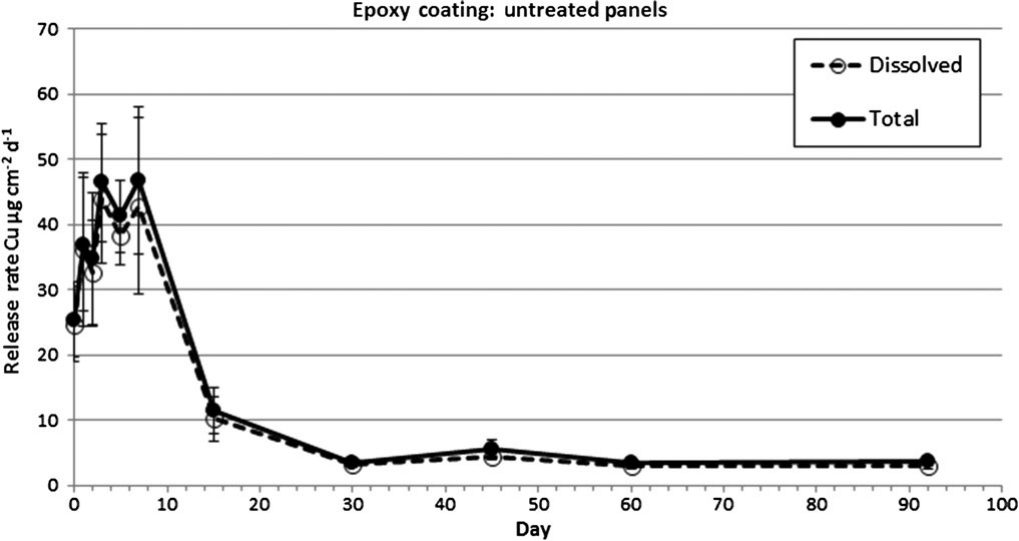
Dissolved and total copper release rates from untreated epoxy-coated panels measured by the in situ dome method.

Following IE, release rates from the untreated panels for both the epoxy and ablative paints measured using the in situ dome method are shown in Table 1. The epoxy coating on the untreated panels showed an initial increase in dissolved copper release rates from day zero through day three from 24.7 to 43.9 μg cm^−2^ d^−1^ (Figure 3). Release rates remained at peak values until day 7 and then rapidly declined between days 7 and 15, when rates rapidly dropped from 42.9 μg cm^−2^ d^−1^ on day 7 to 10.2 μg cm^−2^ d^−1^ on day 15. Values continued to decline through day 30 to 3.1 μg cm^−2^ d^−1^. Release rates appear to reach a PSS from about day 30 through day 92 where values remained stable between 3.1 and 4.5 μg cm^−2^ d^−1^. The calculated average PSS release rate for the replicate panels from day 30 through 92 for the epoxy coating was 3.36 and 4.01 μg cm^−2^ d^−1^, dissolved and total copper, respectively. The ratio of dissolved to total copper release rates was similar across all sampling days, with a mean dissolved:total ratio of 0.91.

**Table 1.** Dissolved and total copper release rates from the untreated epoxy and ablative panels.

	Release rate μg Cu cm^−2^ d^−1^							
	Epoxy untreated				Ablative untreated			
	Dissolved		Total		Dissolved		Total	
Day	Mean	Stdev	Mean	Stdev	Mean	Stdev	Mean	Stdev
0	24.7	5.77	25.4	5.77	9.18	0.69	11.3	2.12
1	36.1	11.8	37.0	10.2	20.6	3.54	21.2	2.92
2	32.6	8.0	34.7	10.2	35.6	17.1	37.4	17.7
3	43.9	9.9	46.4	9.03	46.6	16.3	50.6	18.7
5	38.2	4.42	41.3	5.54	43.7	7.77	44.1	5.56
7	42.9	13.6	46.7	11.3	37.3	1.21	39.1	2.32
15	10.2	3.44	11.5	3.60	11.1	4.41	13.3	6.06
30	3.09	0.39	3.37	0.31	3.02	0.5	3.97	1.62
45	4.47	0.47	5.65	1.31	3.47	0.98	4.21	1.10
60	2.95	0.46	3.45	0.80	3.70	1.08	4.02	0.84
92	3.05	0.44	3.59	0.70	3.00	0.62	4.39	0.5

Note: Release rates from day 0 were initiated 1 h after initial deployment.

**Figure 4.**
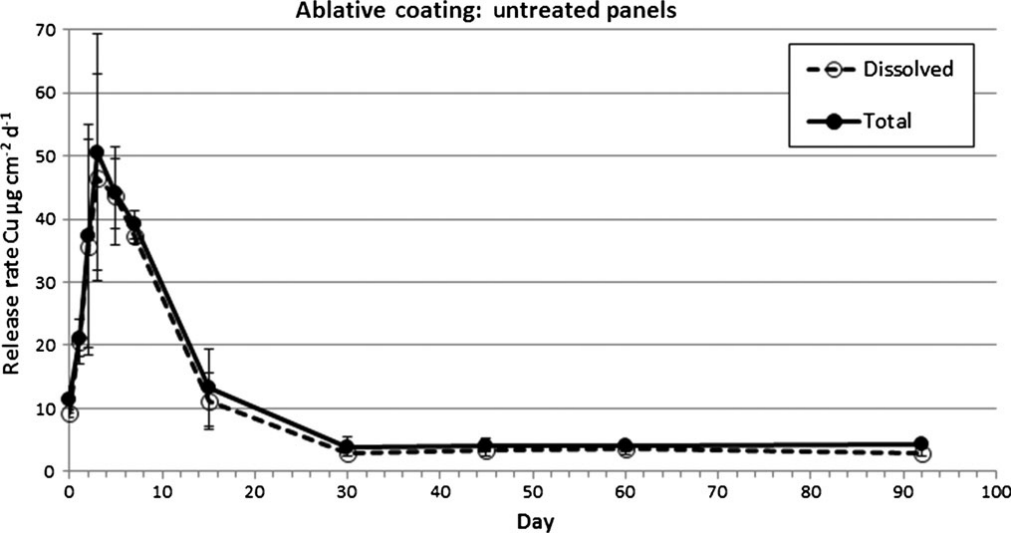
Dissolved and total copper release rates from untreated ablative coated panels measured by the in situ dome method.

The untreated panels for the ablative coating exhibited a similar release rate pattern. There was an initial increase in dissolved copper release rates from day zero through day three from 9.2 to 46.6 μg cm^−2^ d^−1^ (Figure 4). Release rates then began a steady decline to 11.1 μg cm^−2^ d^−1^ on day 15. Values continued to decline through day 30 to 3.0 μg cm^−2^ d^−1^, after which a PSS was achieved. Release rates from day 30 to day 92 were between 3.0 and 3.7 μg cm^−2^ d^−1^. The calculated average PSS release rate for the replicate panels from day 30 through day 92 for the ablative coating was 3.38 and 4.16 μg cm^−2^ d^−1^, dissolved and total copper, respectively. The ratio of dissolved to total copper release rates was similar across all sampling days, with a mean dissolved/total ratio of 0.88.

#### Leachate (L)

**Figure 5.**
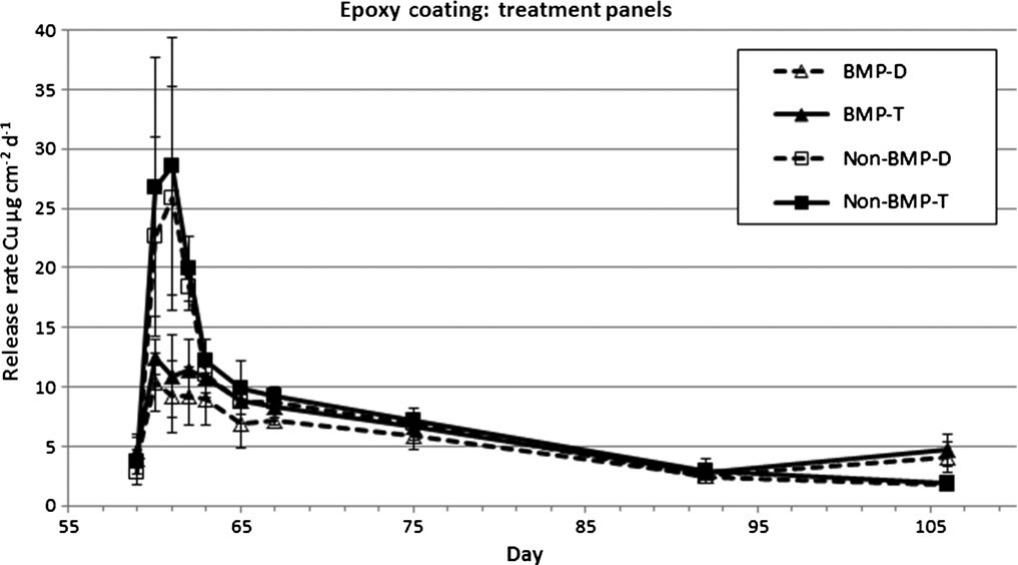
Release rates from BMPs and non-BMP treated epoxy coated panels measured using the in situ dome method. Dissolved and total copper concentrations are shown.

**Figure 6.**
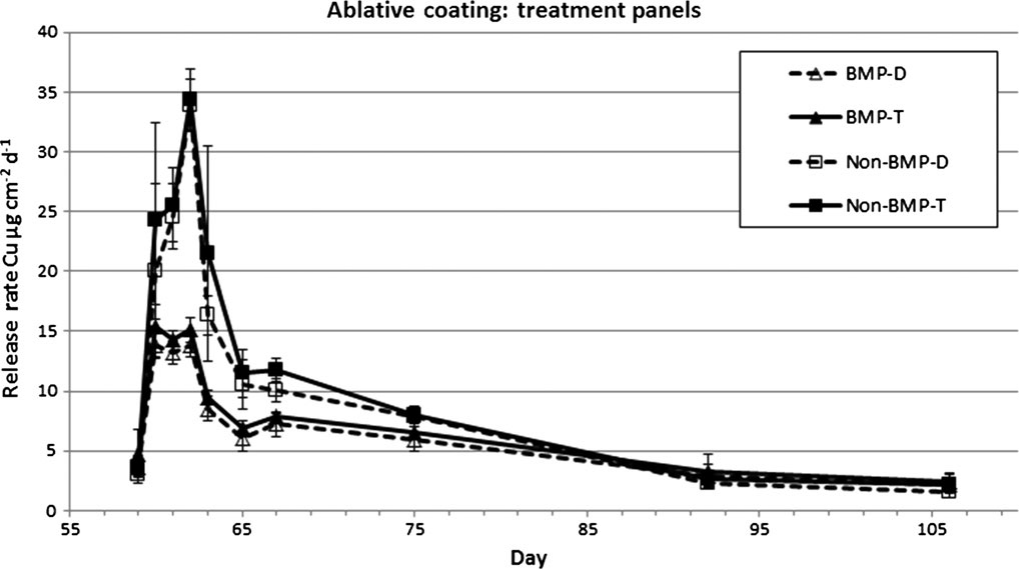
Release rates from BMPs and non-BMP treated ablative coated panels measured using the in situ dome method. Dissolved and total copper concentrations are shown.

Release rates from the treatment panels for both paints, before and after CE (SR_CE_), are shown in Table 2. Dissolved copper release rates measured before the CE on treatment panels on day 59 for both paints were between 2.8 and 4.0 μg cm^−2^ d^−1^. T-test comparison to data from untreated panel release rates on day 60 showed no significant difference between the sets of panels. Following the CE on day 60, the BMP and non-BMP treated panels exhibited similar trends in release rates as the untreated panels for their IE. Total copper release rates showed an initial increase during the first 2– 3 days following the CE, with a drop back to PSS approximately 30 days post cleaning (Figures 5 and 6). The magnitude of the release rates differed between the BMP and non-BMP test treatments for both paint types. The epoxy paint had peak dissolved release rates of 25.8 and 10.4 μg cm^−2^ d^−1^ for the non-BMP and BMP paints, respectively. The ablative paint had peak release rates of 33.9 and 13.9 μg cm^−2^ d^−1^ for the non-BMP and BMP paints, respectively.

**Table 2.** Dissolved and total copper release rates for the BMP and non-BMP epoxy and ablative panels.

	Release rate μg Cu cm^−2^ d^−1^															
	Epoxy BMP				Epoxy non-BMP				Ablative BMP				Ablative non-BMP			
	Dissolved		Total		Dissolved		Total		Dissolved		Total		Dissolved		Total	
Day	Mean	Stdev	Mean	Stdev	Mean	Stdev	Mean	Stdev	Mean	Stdev	Mean	Stdev	Mean	Stdev	Mean	Stdev
59	3.98	1.76	4.61	1.40	2.82	1.04	3.68	1.05	3.58	1.73	4.77	2.03	3.08	0.80	3.59	0.64
**CE on day 60**																
60	10.4	2.45	12.5	1.48	22.6	8.37	26.8	10.9	13.9	0.84	15.4	1.84	20.0	7.26	24.2	8.15
61	9.19	3.01	10.9	3.45	25.8	9.43	28.5	10.8	13.2	0.44	14.3	0.69	24.6	2.70	25.6	3.11
62	9.18	2.45	11.5	2.48	18.4	1.89	19.9	2.76	13.8	0.40	15.1	1.00	33.9	2.17	34.4	2.57
63	8.97	2.18	10.8	1.73	11.0	1.58	12.2	1.74	8.54	0.12	9.46	0.58	16.4	1.64	21.5	8.99
65	6.90	2.02	8.87	0.69	8.71	1.77	9.92	2.26	6.01	0.50	6.87	0.67	10.5	2.07	11.5	2.03
67	7.16	0.33	8.27	0.61	8.74	0.44	9.25	0.78	7.23	0.53	7.90	0.28	10.1	0.96	11.8	0.93
75	5.91	1.24	6.67	1.19	6.94	0.37	7.17	0.97	6.00	0.30	6.60	0.71	7.91	0.83	7.98	0.72
92	2.51	0.38	2.82	0.40	2.46	0.20	2.90	1.02	2.93	1.34	3.22	1.46	2.33	0.32	2.68	0.69
106	4.09	1.33	4.68	1.39	1.78	0.34	1.95	0.12	2.14	0.68	2.41	0.62	1.53	0.06	2.19	0.50

#### CE total and dissolved mass loading

**Figure 7.**
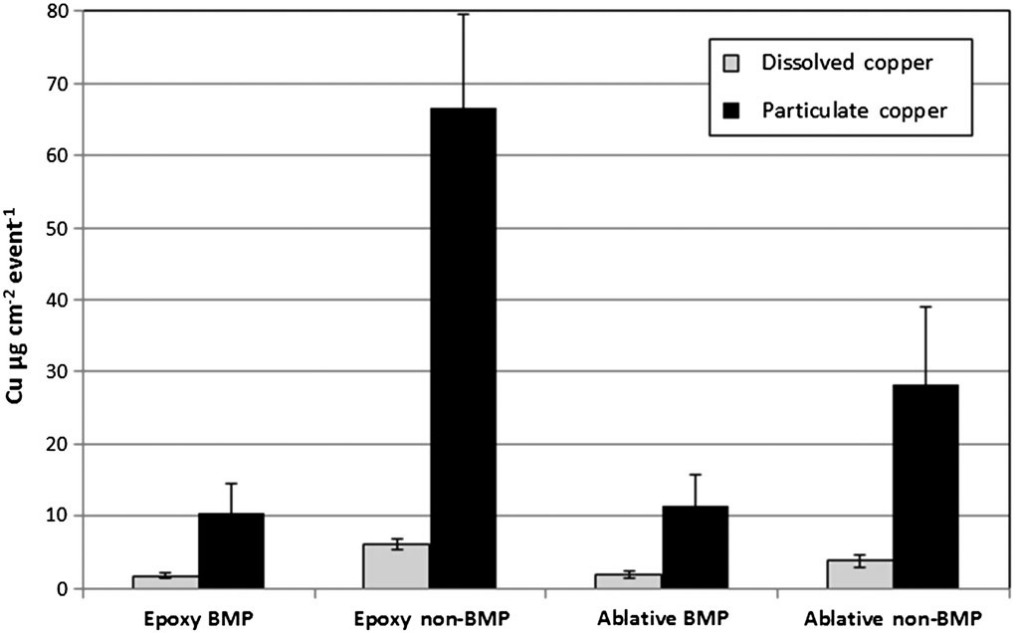
Mass of dissolved and particulate copper (μg cm^−2^ event^−1^) released after the BMPs and non-BMP treatments from the epoxy and ablative coatings. Samples were collected in triplicate with the in-water hull cleaning sampling device from each panel.

Prior to cleaning, all panels were observed to have a thin biofilm layer and microalgal fouling on them with no hard fouling or large macrofauna present. Loading of dissolved and particulate copper due to active cleaning of the biofouling was quantified on day 60 with the in-water hull cleaning sampling method (Chadwick et al. 2008). Procedural blanks indicate a negligible contribution from the cleaning material to the copper load released (Figure 7). Application of the BMP treatment resulted in similar dissolved and particulate mass loading of copper released (μg cm^−2^ event^−1^) from both paints. The mean dissolved copper released was 1.84 and 1.77 μg cm^−2^ event^−1^ for the ablative and epoxy paints, respectively. The release of particulate copper was 11.3 and 10.4 μg cm^−2^ event^−1^ for the ablative and epoxy paints, respectively (Figure 7). For the non-BMP treatment, the mean copper release was 3.84 and 6.06 μg cm^−2^ event^−1^ dissolved and 28.3 and 66.6 μg cm^−2^ event^−1^ particulate for the ablative and epoxy paints, respectively (Figure 7).

### CE speciation studies

Total dissolved copper concentrations determined outside the paint panel area (ambient pier samples) ranged from 12.97 to 22.04 nM (0.82–1.4 μg 1^−1^). The copper-binding ligand concentrations ranged from 15.15 to 81.74 nM (0.96–5.20 μg 1^−1^), comprising 0.02–0.04% of the total DOC pool (Table 3). The strengths of the ambient ligands (conditional stability constant, log K_CuL, Cu2+_^cond^) were very similar on all days sampled, ranging from 12.07 to 12.48. Calculated log[Cu^2+^] values in the ambient samples were below the toxicity threshold for copper (−11) in the marine environment (Brand et al. 1986), with values ranging from −11.35 on day 1 of the sampling to a range of −12.80 to −12.39 on day 60.

**Table 3.** Treatment type, day, dissolved copper concentration (nM), free copper (log[Cu^2+^], ligand concentration (nM), log K_Cul, Cu2+_^cond^ and DOC concentration (μM) data from days 1, 60, and 61.

Treatment	Day	[Cu] nM	log[Cu^2+^]	[L] nM	logK	DOC μM
Ambient	1	14.38	−11.35	15.15	12.26	76.75
A Untreated 1	1	204.5	−11.82	243.3	12.54	76.63
A Untreated 2	1	204.5	−11.07	159.7	13.08	
E Untreated 1	1	604.4	−11.56	621.8	13.08	73.81
E Untreated 2	1	604.4	−10.19	597.1	12.22	
Ambient	60	12.97	−12.80	81.74	12.07	184.0
Ambient	60	14.05	−12.39	25.35	12.48	114.6
Ambient	60	22.04	−12.41	69.44	12.08	115.4
A Untreated 1	60	300.2	−11.46	300.2	14.08	
A Untreated 2	60	307.6	−12.70	326.4	13.91	
E Untreated 1	60	262.7	−11.23	263.3	13.39	
E Untreated 2	60	94.64	−11.98	115.2	12.64	
A BMP 1	60	370.7	−8.98	159.7	11.96	
A BMP 2	60	417.2	−10.45	412.2	12.75	
E BMP 1	60	218.6	−9.85	190.5	12.46	
E BMP 2	60	372.5	−10.33	363.3	13.62	
A non-BMP 1	60	464.7	−9.97	443.9	12.91	
A non-BMP 2	60	599.7	−10.38	592.6	13.05	
E non-BMP 1	60	441.7	−9.80	410.0	12.89	
E non-BMP 2	60	624.3	−9.45	553.3	13.11	
A BMP 1	61	377.7	−9.77	344.0	13.72	
E BMP 1	61	645.2	−9.55	589.1	13.43	
A non-BMP 1	61	267.5	−9.32	287.9	12.45	
E non-BMP 1	61	550.8	−9.21	545.2	13.03	

Notes: Ambient data represent samples collected outside the paint panel area, reflecting ambient copper and copper-binding ligand concentrations in San Diego Bay. Ablative (A) and epoxy (E) represent two treatments, of either the BMP or non-BMP cleaning methods. All samples were taken outside the dome device, except for A and E untreated 2 on day 1.

Samples collected within the study region contained higher concentrations of copper-binding ligands compared to ambient samples. Higher ligand concentrations, and excess ligand (eL) concentrations (defined as [L]–[Cu], Table 3), near the painted panels resulted in free copper concentrations that were relatively similar to the ambient levels on day 1, with log[Cu^2+^] ranging from −11.56 to −11.82 (−11.35 for ambient on day 1). Slightly higher free copper was calculated in samples collected from within the in situ dome on day 1 (−10.19 to −11.07).

**Figure 8.**
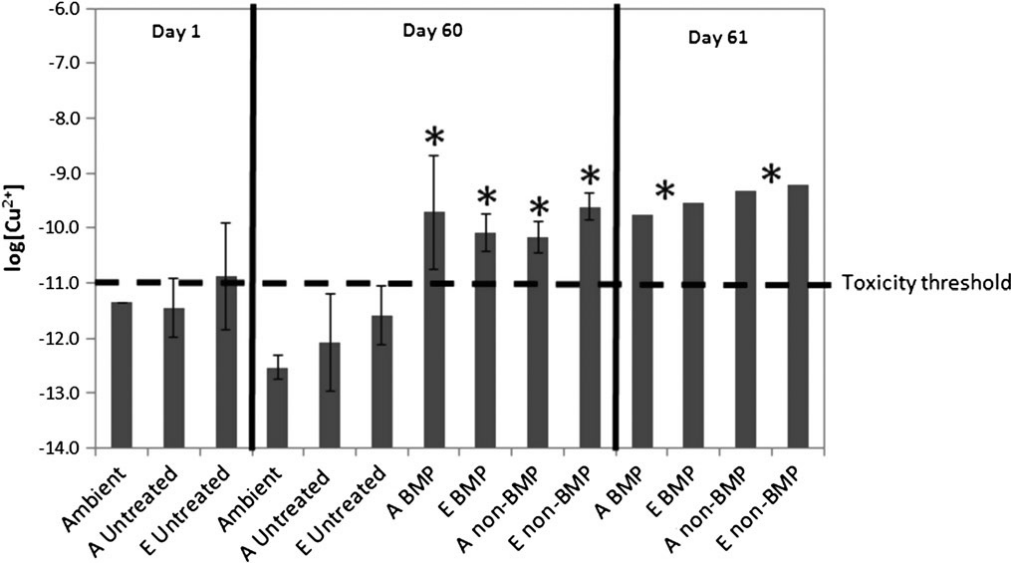
Average free copper (log[Cu^2+^]) concentrations on days 1, 60, and 61 (n = 1 on day 1 for the ambient and on day 61) in all treatments. The dashed line denotes the toxicity threshold for free copper in the marine environment (Brand et al. 1986). The *denotes statistical significance from the ambient for that sampling day at the 95% confidence interval (t-test), except in the case of day 61 when no ambient sample was taken. BMP and non-BMP denotes the BMPs, A denotes ablative coating, and E denotes epoxy coating.

On day 60, the ablative and epoxy-painted controls displayed a similar range of free copper concentrations (−11.23 to −12.70) as on day 1 (−11.56 to −11.82), below the toxicity threshold for copper (t-test, p < 0.05; Table 3, Figure 8). After the CE, free copper concentrations increased for the treated panels on day 60, with all treatments exceeding the toxicity threshold for copper (Table 3, Figure 8). The average log[Cu^2+^] concentrations in the two non-BMP treatments were significantly different from ambient samples on day 60 (t-test, p < 0.002) with average log[Cu^2+^] equal to −10.18 ± 0.29 and –9.62 ± 0.25 in the ablative and epoxy treatments, respectively (Figure 8). The BMP panels on average contained significantly elevated free copper concentrations compared to ambient samples (t-test, p < 0.02), with average free copper concentrations in the ablative treatment equal to –9.71 ± 1.04 and −10.09 ± 0.34 in the epoxy paint treatment (Figure 8). Dissolved copper concentrations exceeded ligand concentrations (negative excess ligand concentrations, Table 3) in the treated panels, indicating the ligand pool was completely titrated with copper in those treatments, leading to the high [Cu^2+^] observed.

Similar results to those observed after the CE on day 60 were obtained on day 61, 24 h after the CE (Table 3). The free copper concentrations calculated on day 61 in each of the treatments were within the range observed on day 60. These concentrations of Cu^2+^ were significantly different from the average ambient free copper concentrations observed on day 60 (t-test, p < 0.002; Figure 8). Ligand concentrations were lower than the dissolved copper concentrations in all treatments on day 61, contributing to the high free copper concentrations calculated on that day (Table 3).

### Life cycle loading estimates

Cumulative release was calculated for the input variables used to estimate life cycle loading over a three-year period (Table 4). During the initial paint exposure period of 60 days, the epoxy coating released 690 μg cm^−2^ dissolved copper and 763 μg cm^−2^ of total copper, while the ablative coating released 657 μg cm^−2^ dissolved copper and 730 μg cm^−2^ total copper. The hull leachate summer and non-summer variables were calculated using the cumulative release over 21 and 28 days, respectively, following the CE on day 60, equating to days 81 and 88, respectively. Release rate measurements were not taken at these specific time intervals, but rather on days 75 and 92. Release rates for days 81 and 88 were extrapolated from the day 75 and 92 data. Hull leachate during the summer cleaning scenario (L_CE21_) for the epoxy coating released 142 μg cm^−2^ dissolved copper (166 μg cm^−2^ total) using the BMP method, and 197 μg cm^−2^ dissolved copper (213 μg cm^−2^ total) was released using the non-BMP method. The L_CE21_ values for the ablative coating were 151 μg cm^−2^ dissolved copper (167 μg cm^−2^ total) using the BMP method and 237 μg cm^−2^ dissolved copper (260 μg cm^−2^ total) using the non-BMP method. Corresponding values for the L_CE28_ input can be found in Table 4.

**Table 4.** Calculation of input variables in life cycle loading.

		Epoxy paint		Ablative paint	
Loading scenario	Duration (days)	Dissolved Cu μg cm^−2^	Total Cu μg cm^−2^	Dissolved Cu μg cm^−2^	Total Cu μg cm^−2^
Initial exposure (IE)	60	690	763	657	730
Leachate summer (L_CE21_): BMP	21	142	166	151	167
Leachate summer (L_CE21_): Non-BMP	21	197	213	237	260
Leachate non-summer (L_CE28_): BMP	28	165	192	177	195
Leachate non-summer (L_CE28_): Non-BMP	28	222	240	263	287
Surface refreshment cleaning event (SR_CE_): BMP	N/A	1.77	10.4	1.84	11.3
Surface refreshment cleaning event (SR_CE_): Non-BMP	N/A	6.06	66.6	3.84	28.3
		Cu	Cu	Cu	Cu
		μg cm^−2^ d^−1^	μg cm^−2^ d^−1^	μg cm^−2^ d^−1^	μg cm^−2^ d^−1^
Pseudo-Steady State (PSS)	N/A	3.36	4.01	3.38	4.16

Note: PSS release rates calculated from the untreated panels. PSS rates were calculated from released rates measured on days 30 through 92.

**Figure 9.**
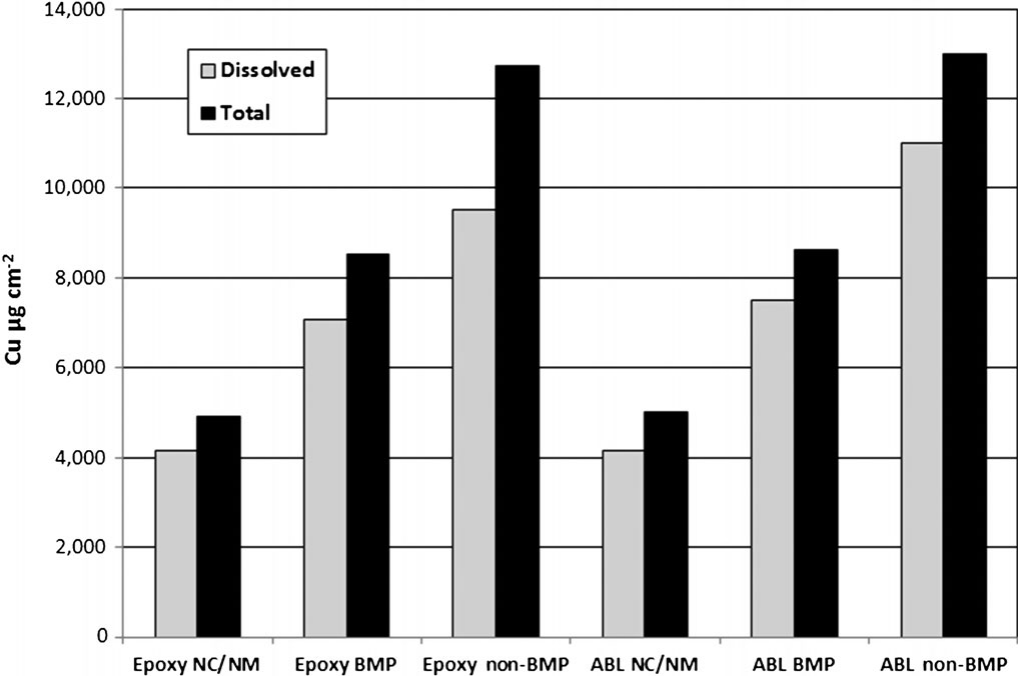
Life cycle loading values for the epoxy and ablative coatings under different cleaning scenarios. NC/NM = no cleaning/no movement. ABL = ablative coating.

Estimated life cycle loading values based on a three-year period are presented in Table 5 and in Figure 9. The dissolved copper loading under the no cleaning/no usage scenario was 4170 μg cm^−2^ for the epoxy coating and 4157 μg cm^−2^ for the ablative coating. Although this is an unrealistic representation of loading, it provides the minimum baseline environmental loading associated with each paint. The epoxy coating had an estimated three-year dissolved copper loading of 7084 μg cm^−2^ for the BMP treatment and 9517 μg cm^−2^ for the non-BMP treatment. The ablative coating had an estimated three-year dissolved copper loading of 7504 μg cm^−2^ for the BMP and 11,023 μg cm^−2^ for the non-BMP. For the epoxy coating, utilizing the BMP vs non-BMP resulted in a reduction of 26% dissolved copper loading and a 33% lower total copper loading than the non-BMP. For the ablative coating, utilizing the BMP methodology resulted in a 32% lower dissolved copper loading and a 34% lower total copper loading than the non-BMP.

**Table 5.** Estimates of life cycle loading for epoxy and ablative coatings using no cleaning/no movement, BMP and non-BMP scenarios.

		No cleaning/no movement		BMP		Non-BMP	
Coating	3 year life cycle loading	Dissolved Cu μg cm^−2^	Total Cu μg cm^−2^	Dissolved Cu μg cm^−2^	Total Cu μg cm^−2^	Dissolved Cu μg cm^−2^	Total Cu μg cm^−2^
Epoxy	Leachate	4,170	4,913	7,013	8,136	9,274	10,037
	Cleaning	–	–	71	416	242	2,664
	Total	4,170	4,913	7,084	8,552	9,517	12,701
	% load from cleaning	0%	0%	1%	5%	3%	21%
Ablative	Leachate	4,157	5,030	7,431	8,188	10,870	11,885
	Cleaning	–	–	74	452	154	1132
	Total	4,157	5,030	7,504	8,640	11,023	13,017
	% load from cleaning	0%	0%	1%	5%	1%	9%

Note: Values calculated from Equation 4.

## Discussion

Copper release rates measured in this study had similar temporal trends compared to previous paint exposure experiments (Seligman et al. 2001; Valkirs et al. 2003; ASTM 2006; ISO 2007b). There was an initial increase in copper release for three days immediately following exposure to seawater, followed by a rapid decrease in release rates for about four days, ultimately reaching an asymptotic PSS value after about 30 days. This pattern was observed for both the epoxy and ablative paints during the IE as well as following the BMP (soft pile carpet) and non-BMP (3M™ Pad) CEs. Peak release rates differed between the various loading scenarios with IE having the highest of about 50 μg cm^−2^ day^−1^, followed by non-BMP treatment at ∼ 35 μg cm^−2^ day^−1^, and the BMP treatment having the lowest at ∼ 10 μg cm^−2^ day^−1^. Although the magnitude of copper release varied, the time to reach a PSS appears to be similar. For both paints and all three loading scenarios, a PSS appears to be reached about 30 days post event. Peak release rates for both the epoxy and ablative coating were ∼ 2.5× for the non-BMP vs the BMP method. This difference translates into cumulative dissolved copper loading during a three-week summer cleaning cycle of 135 μg cm^−2^ (BMP) and 189 μg cm^−2^ (non-BMP) for the epoxy coating, and 146 μg cm^−2^ (BMP) and 227 μg cm^−2^ (non-BMP) for the ablative coating. Although no measurements of release rate were made related to dynamic panel movement (ie simulated boat operation) causing SR, Lindner (1993) found similar release rate trends, in terms of magnitude and periodicity, as those observed with the CE in this study.

*In situ* leach rate results from this study were similar to past research results examining ablative and epoxy AF paints utilizing the dome method (Seligman et al. 2001; Valkirs et al. 2003; Schiff et al. 2004). Schiff et al. (2004) presented release rate data for two copper-based AF coatings and a biocide-free coating over a 28-day study. Seligman et al. (2001) and Valkirs et al. (2003) looked at static and dynamic release rates of nine copper-based AF coatings under laboratory and in situ conditions over two years. Focusing on the epoxy-based coating common to all studies, Schiff et al. (2004) found peak copper release rates observed one day after cleaning of ∼ 18 μg cm^−2^ d^−1^ for an epoxy based paint. Seligman et al. (2001) and Valkirs et al. (2003) reported peak release rates ranging from ∼ 18 to 30 μg cm^−2^ d^−1^ for an ablative coating measured on various test panels. The results presented herein fall within the range of previously reported data with peak dissolved copper release rates ranging from ∼ 10 to 47 μg cm^−2^ d^−1^ following initial paint exposure or the CE, depending on coating type and cleaning method. Lindner (1993), Seligman et al. (2001) and Valkirs et al. (2003) showed that release rates due to dynamic exposures were similar in magnitude to release rates following initial paint exposure. Therefore, it is reasonable to assume that the release rates following SR due to CEs would be similar in magnitude to release rates measured following either dynamic refreshment or vessel movement. The SR capacity of a coating is assumed to be an inherent function of the paint and subsequent copper release is tied to cleaning schedule, BMP method selection, and boat usage. This study additionally sought to gain a better resolution of copper release rates during the most volatile timeframe of the first few days after exposure or SR by measuring leach rates from triplicate panels as well as including time points 1 h after exposure and on days one, two, and three, etc. Immediately following paint exposure or SR, there appeared to be inherent variability associated with paint acclimation as indicated by the large SD within replicate samples during this time. This variability may be due to small-scale differences in biofilm development, as initial surface colonizing bacteria are reported to be highly active during the first 72 h of exposure (Dang & Lovell 2000; Stoodley et al. 2002; Jones et al. 2007). The physico-chemical surface properties of the coating become modified as the biofilm develops (Dang et al. 2008). This rapid change in surface properties may affect copper release. As the microbial biofilm becomes more established, the inter-replicate variability decreases. The rate at which initial colonization takes place may drive replicate variability during the initial week of exposure.

Copper release from the CE was consistent with previous studies that investigated loading related to underwater hull cleaning using BMP and non-BMP methods (Schiff et al. 2004; Port of San Diego 2006). Schiff et al. (2004) presented estimates of copper release rates following BMP and non-BMP methods for epoxy and hard vinyl coatings. Panels were placed in a plastic bin, cleaned, and then removed. Particulate matter within the bin was allowed to settle for 1 h and then a water sample was collected for dissolved copper. Schiff et al. (2004) do not provide an estimate of total copper concentrations associated with particle release during the cleaning. The Port of San Diego (2006) study estimated dissolved and particulate copper release from common in-water hull cleaning practices. The study design for the Port paper included three AF coatings (epoxy based, vinyl-based, and ablative) with three different cleaning materials (soft carpet, medium scour pad, and moderately aggressive nylon brush) at two time intervals, one month and three months. The cleaning device and process in the present study was identical to the Port of San Diego (2006) study, with the exception that the Port study obtained samples directly from boat hulls having variable aged paints while this study employed newly painted panels. Focusing on the epoxy coating common to all three studies, the data presented herein are in closer agreement to those found in the Port study. Dissolved copper concentrations reported in the present study were 1.77 μg cm^−2^ event^−1^ for the BMP and 6.06 μg cm^−2^ event^−1^ for non-BMP. The Port study reports 3.8 μg cm^−2^ per event for the BMP and 8.1 μg cm^−2^ per event for the non-BMP after one month, while Schiff et al. (2004) report 8.57 and 17.45 μg cm^−2^ event^−1^ for the BMP and non-BMP, respectively. Data obtained following the three-month cleaning reported in Port study where only slightly higher at 3.9 (BMP) and 10.5 (non-BMP) μg cm^−2^ event^−1^ dissolved copper. The higher values reported by Schiff et al. (2004) are most likely due to differences in cleaning/sampling methodologies. The present study and the Port study both collected samples immediately following a cleaning, while Schiff et al. (2004) waited an hour after sampling when removed particles may have continued to leach copper into the collection vessel holding seawater. Additionally, in terms of true CE contribution, the Schiff et al. (2004) estimates do not take into account the total particulate load, only dissolved copper. Particulate copper emissions from the present study were in agreement with results reported from the Port study (2006). The results indicate total particulate copper released during the CE from the epoxy coating was 10.4 and 66.6 μg cm^−2^ event^−1^, for the BMP and non-BMP, respectively. Whereas, the Port study reports 8.9 and 47.2 μg cm^−2^ event^−1^, for the BMP and non-BMP, respectively, at one month, and 13.4 and 62.1 μg cm^−2^ event^−1^ for the BMP and non-BMP, respectively, at three months.

During the CE, the mass of dissolved and particulate copper released was dependent on the cleaning procedure. In the case of the BMP scenario, the mass of copper released was identical for the two AF coatings tested with a mean of 1.81 μg cm^−2^ event^−1^ dissolved and 11 μg cm^−2^ event^−1^ particulate. In contrast, when the non-BMP treatment was applied, the amount of dissolved and particulate copper released varied between the two coating types. The epoxy paint released 6.06 μg cm^−2^ event^−1^ dissolved, with a particulate release of 66.6 μg cm^−2^ event^−1^, while the ablative paint released 3.84 μg cm^−2^ event^−1^ dissolved copper and 28.3 μg cm^−2^ event^−1^ particulate copper (Figure 7). The BMP practice is meant to lessen impacts to the paint surface and reduce copper loading by minimizing particulate release. The low values and similarity within the dissolved and particulate releases observed with the BMP procedure suggest that these loading values may be representative of the copper concentration in the biofilm on the coating. In contrast, the larger values and greater disparity between the dissolved and particulate releases measured with the non-BMP treatment suggest an additional loading contribution directly from the coating. The differences between total particle loads from the two paint types may be explained by characteristics inherent to the function of ablative and epoxy-based coatings that utilize different physical and mechanical properties to release copper to reduce biofouling. Additionally, the copper content in the epoxy coating was comprised of 68% cuprous oxide, whereas the ablative paint had a cuprous oxide content of 38%. The results of this study suggest that the BMP cleaning method mostly affects the biofilm and growth on the surface of the coating, while the non-BMP method abrades into the AF paint itself.

### Dissolved, total, and free copper [Cu^2+^]

Dissolved and total copper were measured throughout the present study and were found to be closely related across the sample analyses. Most of the following discussion points are focused on the dissolved fraction of the metal because it more closely approximates the bioavailable fraction of metal in the water column than the total fraction (USEPA 1993). A significant difference between the total and dissolved copper measurement was associated with the CEs. Considering the three-year scenario, the copper loading associated with BMP CEs for both epoxy and ablative paints contributed ∼ 5% of the overall particulate (total) and 1% of the overall dissolved copper. For non-BMP CEs, the loading values for the two paints were different. The overall copper loading associated with the ablative paint contributed ∼ 9% particulate (total) and 1% dissolved copper, and for the epoxy paint the overall copper loading was 21% particulate (total) and the dissolved loading was 3% copper (Table 5). These data show that the individual BMPs and type of paint have significant impacts on the overall environmental loading. The use of BMPs contributes one third of the amount of particulate loading compared to the use of non-BMPs.

It is important to take into consideration that the release of copper, either in the dissolved or particulate phases, to the marine environment is subject to complex environmental processes (Morel 1983; Rivera-Duarte & Flegal 1997). Depending on the size of the particles, particulate copper will be affected by dissolution processes, but particles should fall through the water column relatively quickly and settle on the surface of the sediment. Dissolved copper released from the paints should remain longer in the water column, where it will speciate according to ambient conditions. The chemical speciation theory in general and the free ion model (Morel & Hering 1993) in particular describe the dynamics of copper distribution in natural marine waters. This dynamic equilibrium is important in defining those chemical species of copper that are available to organisms, ie bioavailable. The dissolved copper fraction is differentiated between that which is bound to organic (Campos & van den Berg 1994) and inorganic complexes, colloids, and the free copper ion (Cu^2+^). The free ion model predicts that Cu^2+^ is the species that is bioavailable, and is a better predictor of toxicity. In the ambient samples collected outside the immediate area of this study, the majority of the dissolved copper was found to be associated with strong organic ligands leading to free copper concentrations that were low (Table 3). On day 1 following the IE, free copper also was found to be low in the ablative and epoxy untreated panels despite elevated dissolved copper concentrations (Table 3), presumably due to a source of copper-binding ligands from the biofilm on the AF paint, or from the paint itself. All samples from inside the study area contained much higher ligand concentrations than nearby ambient samples providing strong evidence of copper-binding ligands associated with the paints themselves. It is speculated that the leached copper on day 1 existed as an inorganic copper oxide complex, which could be detected as a ligand with the method employed in this study.

On day 60, immediately following the CE, dissolved copper concentrations associated with the BMP and non-BMP treated panels were higher compared to the untreated (no cleaning) samples. This resulted in significantly elevated free copper concentrations for the treatment panels compared to untreated panels (Figure 8). Thus, in terms of the toxicity of copper as interpreted by the concentration of Cu^2+^, cleaning appears to have more of an effect than initial paint exposure, despite the lower dissolved copper release rates associated with cleaning vs IE. This observation is reinforced by the data from day 61 (one day following the CE), where release rates were somewhat elevated compared to the those 1 h after the CE (Table 2), yet the free copper concentrations were not significantly higher (Figure 8). The data show that copper released during CEs can cause periodic toxicity that may persist until the free copper ion concentrations drop back down to ambient conditions.

### Typical loading scenario

In order to provide context to harbor loading scenarios, the three-year copper loading from values in Table 5 were multiplied by the wetted hull surface area of a typical recreational boat. Formulae vary for the calculation of the wetted hull surface area of a boat or the surface area of AF paint normally submerged in the seawater. A standard estimate used by paint manufacturers for wetted hull surface area that considers average boat shapes was applied as a factor of length (L) by beam width (B) by a standard conversion factor (0.85). A 40 foot long boat with a beam of 13 feet yields a wetted hull surface of 442 ft^2^ (410,631 cm^2^).

For a typical 40 foot recreational boat, the average annual loading (based on the three year cumulative loading) ranged from 970 to 1181 g dissolved Cu yr^−1^ for epoxy and ablative paints, respectively, exclusively utilizing the recommended BMP. For use of non-BMP methods, the average annual loading was 1303 or 1780 g dissolved Cu yr^−1^ for epoxy and ablative paints, respectively. Under the no cleaning/no use scenario, a 40 foot recreational boat would release 571 g dissolved Cu yr^−1^ for the epoxy and 569 g dissolved Cu yr^−1^ for the ablative, representing the minimum loading for this vessel. These results are less than other methods for calculating loading estimates such as values extrapolated from ASTM or ISO test results; however, they are based on in situ measurements and are considered more representative of an environmentally relevant loading scenario (Finnie 2006; OECD 2012). The use of BMP values are more appropriate for cumulative loading estimates based on current practices. In general, boat owners and the professional divers/cleaners tend to favor methods that remove fouling in the least damaging manner so that the coating system lasts longer (CPDA 2008). In addition, loading assumptions made for the entire three-year period are conservatively high because they are based on the behavior of the paint system during the first 60 days and only the first CE and do not account for reduced paint efficacy (and lower loading) over the estimated three-year life cycle. The use of non-BMP loading values for a three-year cumulative release is not appropriate based on real-world practices and general BMP implementation, and would, therefore, result in an unrealistic overestimate of loading.

Lindner (1993) examined the long-term behavior of copper-based AF paints that were subject to static and dynamic cycles of exposure. The evaluation included epoxy and ablative AF paints similar to those used in this study and concluded that both paints performed well over a four- to five-year timeframe. Of note is that the Lindner (1993) study did not include any cleaning activities on the coatings. The complicating factor to understand fouling and its impacts on a vessel can vary substantially based on the type and function of a vessel and, more importantly, the opinion of the vessel owner. Human factors are the driving force behind the overall loading calculations and are the ones that cannot be adjusted or accounted for unless they are widespread practices. For example, a pleasure craft with incipient slime on it may be considered fouled by the owner who may insist on cleaning, but it may not impact the overall performance of that particular vessel. However, if the boat is in a race, the owner may feel that any possible impediment to hydrodynamic flow is unacceptable. As a result, the present study used the current status quo associated with typical boat cleaning activities (Port of San Diego 2011).

## Conclusion

This study helps to quantify some of the complicated variables associated with environmental loading parameters from typical recreational boat paints. The selection and use of BMPs for maintaining AF coatings can have a substantial impact on these loading values. On average, the use of BMPs resulted in one-third less copper loading than non-BMP practices. This difference is magnified when extrapolations are made using resident boat populations in a given geographical area.

The relationship of potential ambient toxicity to the use and maintenance of copper-based AF paints indicates that initial panel deployment (eg newly painted) does not exceed toxicity thresholds. However, cleaning activities (regardless of method) result in a greater toxicity potential than initial paint exposure despite the lower dissolved copper release rates associated with cleaning vs IEs.

Loading contributions from copper-based AF paints were quantified for typical boat painting and maintenance activities, excluding any boat use or variable human factors. The life cycle loading formula presented herein can be universally applied to any AF paint, regardless of formulation, to estimate environmental contributions. Future studies to refine loading estimates for AF paints could include variable aged paints and factors associated with vessel use.
